# The complete mitochondrial genome of the terebellid polychaete *Thelepus plagiostoma* (Terebellida; Terebellidae)

**DOI:** 10.1080/23802359.2021.1975510

**Published:** 2021-10-05

**Authors:** Sang-Eun Nam, Hyoung Sook Park, Sung Ah Kim, Bo-Mi Kim, Jae-Sung Rhee

**Affiliations:** a Department of Marine Science, College of Natural Sciences, Incheon National University, Incheon, South Korea; b Department of Song-Do Bio-Environmental Engineering, Incheon Jaeneung University, Incheon, South Korea; c Research Unit of Cryogenic Novel Material, Korea Polar Research Institute, Incheon, South Korea; d Yellow Sea Research Institute, Incheon, South Korea

**Keywords:** Complete mitogenome, Terebellida, Terebellidae, *Thelepus plagiostoma*, polychaete

## Abstract

Here, we report the complete mitogenome information of the terebellid polychaete, *Thelepus plagiostoma* (Schmarda, 1861). Genome sequencing by Illumina HiSeq platform permitted assembly of a circular mitochondrial genome of 15,628 bp from *T. plagiostoma* consisting of 67% AT nucleotides, 13 protein-coding genes (PCGs), 2 ribosomal RNA (rRNA) genes, 22 transfer RNA (tRNA) genes, and a non-coding region in the typical annelid gene composition. Gene order of the *T. plagiostoma* mitochondrion is identical to those of the Terebelliformia mitogenomes. Phylogenetic reconstruction places *T. plagiostoma* within the monophyletic subclass Sedentaria, a sister to *Pista cristata* in the suborder Terebelliformia.

Annelida, also known as a segmented worm, is a complex phylum that occupies a variety of environments (e.g. marine, terrestrial, and fresh water) with notably derived morphologies, outstanding ecological diversity, and broad life strategies such as deposit/filter feeders, herbivores and/or carnivores (Struck et al. 2011; Andrade et al. 2015). Annelids were traditionally classified into Polychaeta and Clitellata, but molecular phylogenomic approach combined with genomic data and morphological parameters indicated the Polychaeta as a synonym of annelids (Struck et al. 2011, 2017; Weigert et al. 2014). Now, classification of the diversity of Annelida consists of two main clades, Sedentaria and Errantia (Struck et al. 2011; Weigert et al. 2014; Weigert and Bleidorn 2016; Struck 2017). Although the two main monophyletic annelid backbone tree is well-established, many areas of the phylogenetic relationship in Sedentaria are still poorly resolved and there are many challenges needed to be focused on this clade.

The spaghetti worms, Terebellids are tubicolous polychaetes and exhibit a wide geographical distribution, ranging from the North Pole to the Antarctic. Recently, phylogenetic subfamilial status in Terebelliformia was restored by means of a combination of 12,674 orthologous gene information and morphological data (Stiller et al. 2020), whereas several species are still missing due to absence of genomic information and uncertain delineation of lineages. The marine polychaete, *Thelepus plagiostoma* Schmarda, 1861 (Terebellida; Terebellidae) inhabits soft sediments from intertidal to shallow offshore reef with depths between 5 and 400 m. Distribution of *T. plagiostoma* has been mostly reported in tropical Indo-West Pacific Ocean and the Antarctica. However, it was also identified at Arctic-Pacific-boreal and Atlantic-boreal shelf (Leontovich 2011). In this study, a specimen of *T. plagiostoma* was collected from the Beaufort Sea (69°52'N, 139°03'W) in 2017 using a remotely operated underwater vehicle (ROV) belonging to the Monterey Bay Aquarium Research Institute (MBARI). The specimen and DNA was deposited in the Research Institute of Basic Sciences of Incheon National University (Species ID: Annelid-04; Specimen ID: KOPRI-Benthos-26; https://www.inu.ac.kr/user/indexMain.do?siteId=ribs; Dr. Sang-Eun Nam; se_nam2@inu.ac.kr).

Genomic DNA was prepared from a specimen muscle using a DNeasy Blood and Tissue kit (Qiagen, Hilden, Germany) according to the manufacturer’s standard protocol. A fragment library was prepared using the TruSeq DNA Sample Preparation Kit (Illumina, San Diego, CA, USA) as previously described (Nam et al. 2021), before sequencing by Illumina HiSeq sequencer. The sequencing library was prepared by random fragmentation of the DNA sample, followed by 5′ and 3′ adapter ligation. Raw reads were obtained from the sample that passed the quality control check in the Illumina HiSeq platform (Illumina) at Macrogen, Inc. (Seoul, South Korea). Adapter sequences, low quality reads, reads with >10% of unknown bases, and ambiguous bases were removed to obtain high quality assembly. After the quality check process, a total of 24,256,126 filtered reads were obtained from 34,479,378 raw reads. Subsequently, *de novo* assembly was conducted with various k-mers using SPAdes (Bankevich et al. 2012), and a circular contig of the *T. plagiostoma* mitogenome was obtained. The resulting contig consensus sequence was annotated using MITOS2 (Bernt et al. 2013) and tRNAscan-SE 2.0 (Lowe and Eddy 1997). Finally, BLAST searches confirmed the identity of the genes (http://blast.ncbi.nlm.nih.gov).

The *T. plagiostoma* circular 15,628 bp mitogenome (GenBank accession no. MW557377) was composed of nucleotide composition: 30.9% A, 12.0% C, 21.3% G, and 35.8% T. The gene order and composition of the *T. plagiostoma* mitogenome is identical to all known Terebelliformia mitogenomes (Nam et al. 2021). Thirteen *T. plagiostoma* mitochondrial protein-coding genes (PCGs) begin with ATG or ATT start codon and the 22 tRNAs have typical cloverleaf secondary structures. The overall genomic architecture of the *T. plagiostoma* mitochondrion is typical for Terebelliformia (e.g. *Melinna cristata*, *Pista cristata*, *Terebellides stroemii*). We reconstructed a phylogeny using the concatenated set of the whole 13 PCGs of the *T. plagiostoma* mitogenome, 6 published mitogenomes belonging to Sedentaria, 17 mitogenomes involved in Errantia, and two Sipuncula species as outgroups (Figure 1). The *T. plagiostoma* formed a sister group with Terebelliformia such as *Terebellides stroemii* (Terebellida, Trichobranchidae), *Melinna cristata* (Terebellida, Melinnidae), and *Pista cristata* (Terebellida, Terebellidae). Since molecular phylogenetic relationship in families belonging to Sedentaria is still controversial due to limited information of whole mitogenomes, the complete mitogenome sequence of *T. plagiostoma* will serve as an essential resource for understanding the phylogenetic relationship and evolutionary history of Terebelliformia.

**Figure 1.**
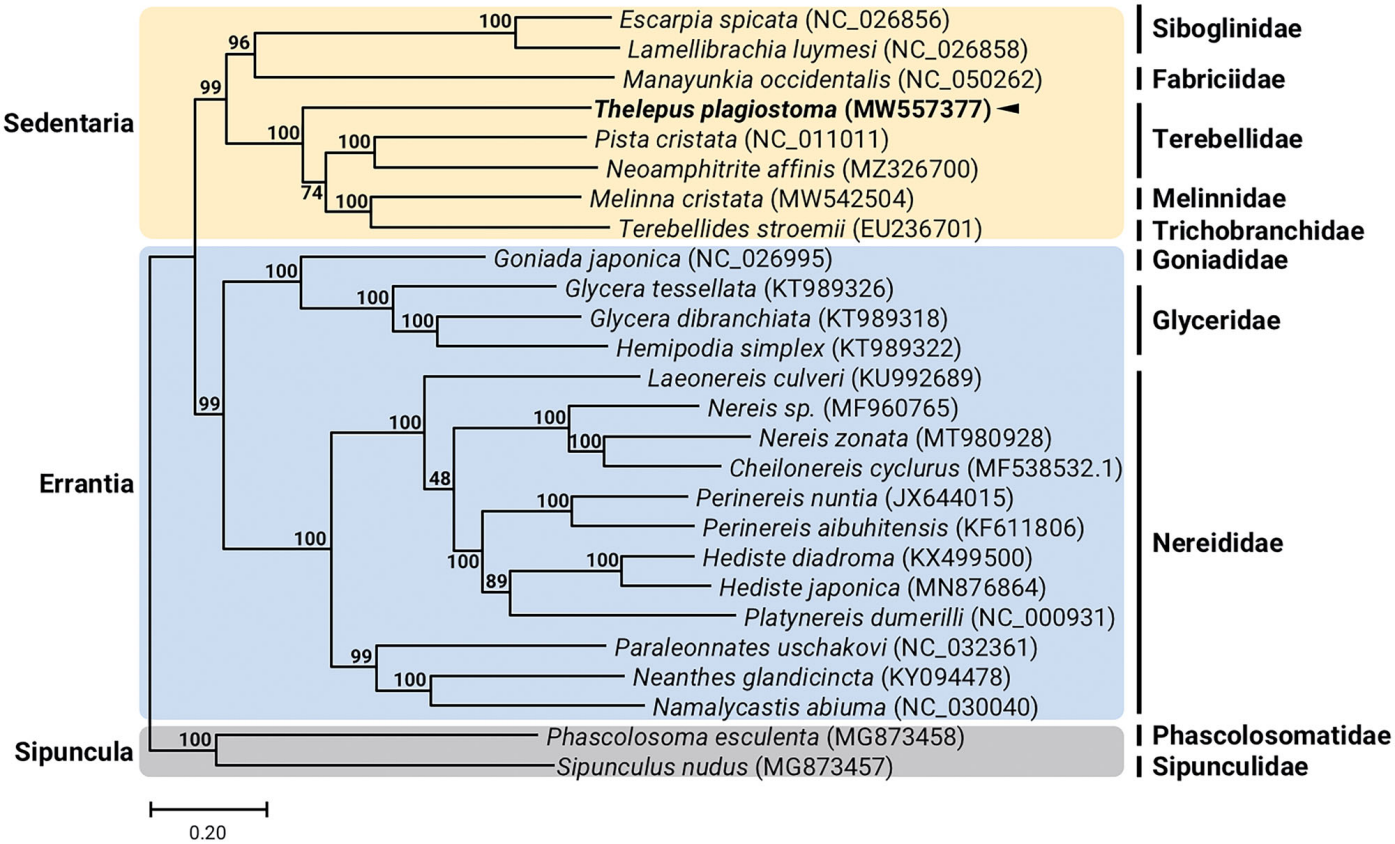
Maximum-likelihood (ML) phylogeny of 7 published mitogenomes from Sedentaria including *T. plagiostoma* and 17 registered mitogenomes of Errantia species, and two Sipuncula species as an outgroup based on the concatenated nucleotide sequences of protein-coding genes (PCGs). The phylogenetic analysis was performed using the maximum likelihood method, GTR + G + I model with a bootstrap of 1,000 replicates. Numbers on the branches indicate ML bootstrap percentages. DDBJ/EMBL/Genbank accession numbers for published sequences are incorporated. The black triangle means the polychaete analyzed in this study.

## Data Availability

BioProject, BioSample, and SRA accession numbers are https://www.ncbi.nlm.ni h.gov/bioproject/PRJNA699104, https://www.ncbi.nlm.nih.gov/biosample/SAMN17766233, and https://www.ncbi.nlm.nih.gov/sra/?term=SRR15041259, respectively. The data that support the findings of this study are openly available in the National Center for Biotechnology Information (NCBI) at https://www.ncbi.nlm.nih.gov, with an accession number MW557377.
